# Antimicrobial Effects of Minocycline, Tigecycline, Ciprofloxacin, and Levofloxacin against *Elizabethkingia anophelis* Using In Vitro Time-Kill Assays and In Vivo Zebrafish Animal Models

**DOI:** 10.3390/antibiotics10030285

**Published:** 2021-03-10

**Authors:** Jiun-Nong Lin, Chung-Hsu Lai, Yi-Han Huang, Chih-Hui Yang

**Affiliations:** 1 Department of Critical Care Medicine, E-Da Hospital, I-Shou University, Kaohsiung 824, Taiwan; 2 Division of Infectious Diseases, Department of Internal Medicine, E-Da Hospital, I-Shou University, Kaohsiung 824, Taiwan; laich6363@yahoo.com.tw; 3 School of Medicine, College of Medicine, I-Shou University, Kaohsiung 824, Taiwan; je091410show@hotmail.com; 4 Department of Biological Science and Technology, Meiho University, Pingtung 912, Taiwan; puppylovefu@gmail.com

**Keywords:** *Elizabethkingia anophelis*, minocycline, tigecycline, ciprofloxacin, levofloxacin, zebrafish

## Abstract

*Elizabethkingia anophelis* is a multidrug-resistant pathogen. This study evaluated the antimicrobial activity of minocycline, tigecycline, ciprofloxacin, and levofloxacin using in vitro time-kill assays and in vivo zebrafish animal models. The *E. anophelis* strain ED853-49 was arbitrarily selected from a bacterial collection which was concomitantly susceptible to minocycline, tigecycline, ciprofloxacin, and levofloxacin. The antibacterial activities of single agents at 0.5–4 × minimum inhibitory concentration (MIC) and dual-agent combinations at 2 × MIC using time-kill assays were investigated. The therapeutic effects of antibiotics in *E. anophelis*-infected zebrafish were examined. Both minocycline and tigecycline demonstrated bacteriostatic effects but no bactericidal effect. Minocycline at concentrations ≥2 × MIC and tigecycline at concentrations ≥3 × MIC exhibited a long-standing inhibitory effect for 48 h. Bactericidal effects were observed at ciprofloxacin and levofloxacin concentrations of ≥3 × MIC within 24 h of initial inoculation. Rapid regrowth of *E. anophelis* occurred after the initial killing phase when ciprofloxacin was used, regardless of the concentration. Levofloxacin treatment at the concentration of ≥2 × MIC consistently resulted in the long-lasting and sustainable inhibition of bacterial growth for 48 h. The addition of minocycline or tigecycline weakened the killing effect of fluoroquinolones during the first 10 h. The minocycline-ciprofloxacin or minocycline–levofloxacin combinations achieved the lowest colony-forming unit counts at 48 h. Zebrafish treated with minocycline or a combination of minocycline and levofloxacin had the highest survival rate (70%). The results of these in vitro and in vivo studies suggest that the combination of minocycline and levofloxacin is the most effective therapy approach for *E. anophelis* infection.

## 1. Introduction

The genus *Elizabethkingia*, which originated from *Flavobacterium* and *Chryseobacterium*, currently comprises six species: *E. meningoseptica*, *E. miricola*, *E. anophelis*, *E. bruuniana*, *E. ursingii*, and *E. occulta* [1]. Bacteria in this genus are aerobic, Gram-negative, glucose-nonfermenting, non-motile, and nonspore-forming bacilli [2]. These microorganisms, particularly *E. anophelis*, occasionally cause life-threatening infections in humans, such as meningitis, nosocomial pneumonia, bacteremia, catheter-related bloodstream infection, urinary tract infection, and biliary tract infection [3,4,5,6,7,8]. Outbreaks of severe *E. anophelis* infections, with an average case fatality rate of 24–60%, have been described in several countries, including Singapore [3], Hong Kong [4], South Korea [5], Taiwan [6,7], and the United States [8].

Numerous studies conducting susceptibility testing using minimum inhibitory concentration (MIC) methods have shown that *E. anophelis* is typically resistant to multiple antibiotics, including β-lactams, carbapenems, β-lactam/β-lactamase inhibitor combinations, aminoglycosides, and colistin [3,4,5,6,7,8]. Almost all *E. anophelis* isolates show susceptibility to minocycline, whereas a wide range of MIC levels have been reported for ciprofloxacin, levofloxacin, and tigecycline [5,6,7]. However, these MIC studies of *E. anophelis* have merely provided static in vitro information on the antimicrobial agents tested; they have not provided kinetic data on the bactericidal rate or activity dosage. At the time of writing this paper, no pharmacodynamic information of antimicrobial agents against *E. anophelis* is available.

The zebrafish (*Danio rerio*) is a tropical freshwater vertebrate that has similar organs and immune system to humans. Many studies have demonstrated that the zebrafish is an excellent and powerful animal model for infectious diseases because of their intact innate and adaptive immune [9]. *Elizabethkingia* species have been known as waterborne pathogens and could infect aquatic animals [10,11,12,13]. Therefore, zebrafish could be a potentially appropriate animal model for *Elizabethkingia* infections.

In this study, we used in vitro time-kill studies to evaluate the antimicrobial effects of minocycline, tigecycline, ciprofloxacin, and levofloxacin against *E. anophelis*, either singly or in combination. We also used zebrafish as an animal model to evaluate the in vivo antimicrobial effects of these tetracycline/glycylcycline and fluoroquinolones in zebrafish animal model with *E. anophelis* infections.

## 2. Materials and Methods

### 2.1. Study Setting and Ethics

The clinical microbiology database of E-Da Hospital, a 1000-bed university-affiliated medical center in Kaohsiung, Taiwan, was searched for routine cultures that yielded *Elizabethkingia* from 2005 to 2019. The collected *Elizabethkingia* isolates were stored as glycerol stocks at −80 °C until use. The precise species of the stored *Elizabethkingia* isolates was re-identified using 16S rRNA gene sequencing, as described in our previous study [6]. This study was approved by the Institutional Review Board of E-Da Hospital. This animal study followed the National Institutes of Health Guide for the Care and Use of Laboratory Animals and was approved by the Institutional Animal Care and Use Committee of E-Da Hospital.

### 2.2. MIC Determination

The MICs of the antibiotics were determined using Sensititre 96-well broth microdilution panels in accordance with the manufacturer’s instructions (Thermo Fisher Scientific/Trek Diagnostics Systems, Oakwood Village, OH, USA). The breakpoints for susceptibility testing were appraised as per the interpretive standards for “other non-*Enterobacteriaceae*” from the CLSI guideline [14]. There are no interpretive criteria for tigecycline against “other non-*Enterobacteriaceae*” from the CLSI guideline. Therefore, the MICs of tigecycline were interpreted according to the *Enterobacteriaceae* susceptibility breakpoints of the US Food and Drug Administration (susceptible MIC ≤ 2 mg/L; intermediate MIC, 4 mg/L; resistant MIC ≥ 8 mg/L) [15].

### 2.3. Bacterial Strain

For in vitro time-kill studies and in vivo animal studies, the MIC results of all collected *E. anophelis* isolates were analyzed. Strain ED853-49 was arbitrarily selected from a collection of *E. anophelis* isolates that had demonstrated susceptibilities to minocycline, tigecycline, ciprofloxacin, and levofloxacin.

### 2.4. In Vitro Time-Kill Studies

The *E. anophelis* strain ED853-49 was cultured overnight at 35 °C in cation-adjusted Mueller–Hinton broth (CAMHB). The bacterial suspension was adjusted to a 1.7 McFarland standard (approximately 5 × 10^8^ colony-forming unit (CFU)/mL). Then, 25 µL of the adjusted bacterial suspension was added to a flask with 25 mL of CAMHB (approximately 5 × 10^5^ CFU/mL). To test the antimicrobial effect of a single antibiotic in time-kill studies, minocycline (Cyrusbioscience Inc., Taipei, Taiwan), tigecycline (Cyrusbioscience), ciprofloxacin (Sigma–Aldrich Inc., St. Louis, MO, USA), and levofloxacin (Sigma-Aldrich) at concentrations of 0.5 ×, 1 ×, 2 ×, 4 × MIC were added to the bacteria-containing flasks at 35 °C for 16–20 h. To investigate the synergistic and antagonistic effects of two antibiotics in time-kill studies, minocycline–ciprofloxacin, minocycline–levofloxacin, tigecycline–ciprofloxacin, and tigecycline–levofloxacin combinations were examined. Each antimicrobial agent at 2 × MIC was used for combination studies because the inhibitory activity against *E. anophelis* persisted for at least 12 h at concentrations equal to two or more times the MIC (shown later in the section of results) [16]. After an incubation time of 0, 2, 4, 6, 8, 10, 24, 30, and 48 h, 50 µL of bacterial suspension was added to 450 µL of PBS. The colony number was counted on Mueller–Hinton agar plates inoculated with 100-µL aliquots of 10-fold serially diluted bacterial suspension after 16–20 h of incubation at 35 °C. All experiments were repeated five times. The highest and lowest values were excluded. The average (mean) of the middle three values was calculated.

### 2.5. Analysis of Time-Kill Curves

The results of time-kill studies were analyzed using a previously reported method [16]. Antimicrobial agents were considered to be bactericidal if a CFU number decrease of ≥3 log_10_ compared with the initial inocula within 24 h [17]. Synergy of a given antimicrobial combination was defined as a CFU number decrease of ≥2 log_10_ compared with the most active single agent, and antagonism was defined as a ≥2 log_10_ increase in the CFU number compared with the most active single antibiotic [17].

### 2.6. Preparation of Bacteria for the Animal Study

The *E. anophelis* strain ED853-49 was overnight cultured in CAMHB at 37 °C. Then, the bacterial suspension was centrifuged at 10,000 g for 3 min. The supernatant of the centrifuged solution was removed, and the left cell pellet was re-suspended in 1 mL of 0.9% saline. These processes were repeated two times. The final bacterial suspension was adjusted to 7.5 × 10^9^ CFU/mL by an OD_600_ spectrophotometer.

### 2.7. Antimicrobial Effects in Zebrafish with E. anophelis Infection

Approximately 7-month-old adult zebrafish of the wild-type AB-line strain were used in this study (G. Fish Animal Model Inc., Taipei, Taiwan). The care, housing, anesthetization, and euthanization of zebrafish were completed as in our previous study [18]. A total of 10 µL of bacterial solution (7.5 × 10^7^ CFU) was injected into the peritoneal cavity of zebrafish as described previously [19]. Each group tested contained ten zebrafish. Antibiotics were given via intraperitoneal injection 2 h after bacterial injection as described previously for the peritonitis mouse model [20,21]. The dosages of single-agent and dual-agent therapy were as follows: Minocycline (Cyrusbioscience), 10 mg/kg every 12 h; tigecycline (Pfizer Inc., New York City, NY, USA), 25 mg/kg every 12 h; ciprofloxacin (Bayer AG, Leverkusen, Germany), 8 mg/kg every 12 h; and levofloxacin (Daiichi Sankyo Inc., Tokyo, Japan), 15 mg/kg every 24 h. The zebrafish were observed for a total of 72 h after antibiotic infections. Kaplan–Meier survival analysis was performed using SPSS version 18.0 (IBM, Armonk, NY, USA). A two-tailed *p* < 0.05 was considered statistically significant.

## 3. Results

### 3.1. Antimicrobial Susceptibility Determined Using MIC

The E. anophelis strain ED853-49 was resistant to piperacillin (MIC = 256 mg/L), piperacillin–tazobactam (MIC = 256/4 mg/L), ticarcillin–clavulanate (MIC = 256/4 mg/L), ceftazidime (MIC > 256 mg/L), cefepime (MIC > 256 mg/L), gentamicin (MIC > 256 mg/L), amikacin (MIC > 256 mg/L), and imipenem (MIC > 8 mg/L). By contrast, this strain was susceptible to minocycline (MIC = 0.25 mg/L), tigecycline (MIC = 0.5 mg/L), ciprofloxacin (MIC = 0.5 mg/L), and levofloxacin (MIC = 0.25 mg/L).

### 3.2. Time-Kill Studies of Single-Agent Therapy

**Figure 1:**
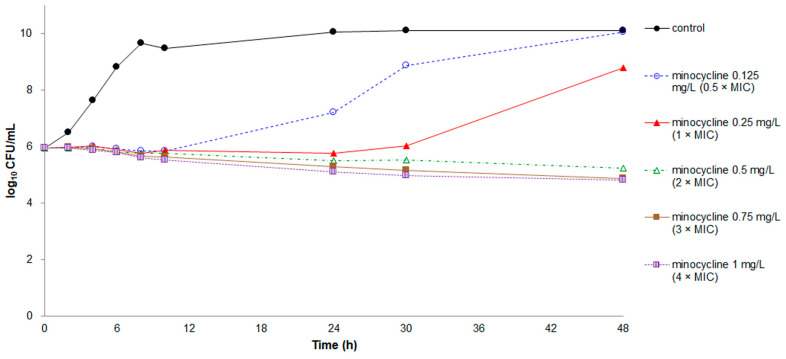
Time-kill assays for minocycline against *Elizabethkingia anophelis* strain ED853-49. Data points represent the mean of three experiments. Minimum inhibitory concentration (MIC) of minocycline = 0.25 mg/L.

**Figure 2:**
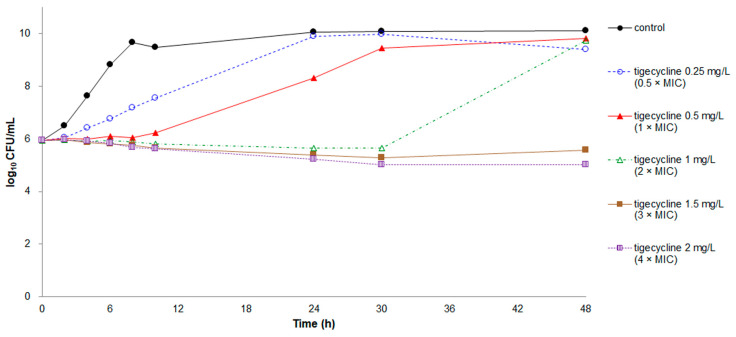
Time-kill assays for tigecycline against *E. anophelis* strain ED853-49. Data points represent the mean of three experiments. MIC of tigecycline = 0.5 mg/L.

The growth patterns and time-kill curves of minocycline and tigecycline were similar (Figure 1 and Figure 2). Poor bacteriostatic effects and regrowth of bacteria were observed in minocycline at concentrations of 0.5–1 × MIC and in tigecycline at concentrations of 0.5–2 × MIC. Only minocycline at concentrations of 2–4 × MIC and tigecycline at concentrations of 3–4 × MIC constantly inhibited bacterial growth for 48 h. However, both minocycline and tigecycline exhibited no bactericidal effect at any concentration.

**Figure 3:**
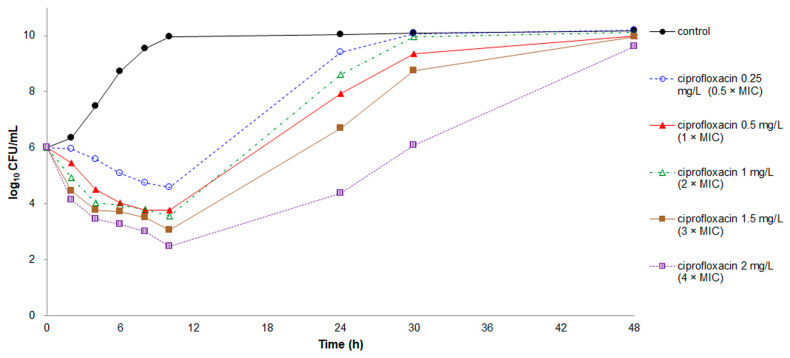
Time-kill assays for ciprofloxacin against *E. anophelis* strain ED853-49. Data points represent the mean of three experiments. MIC of ciprofloxacin = 0.5 mg/L.

**Figure 4:**
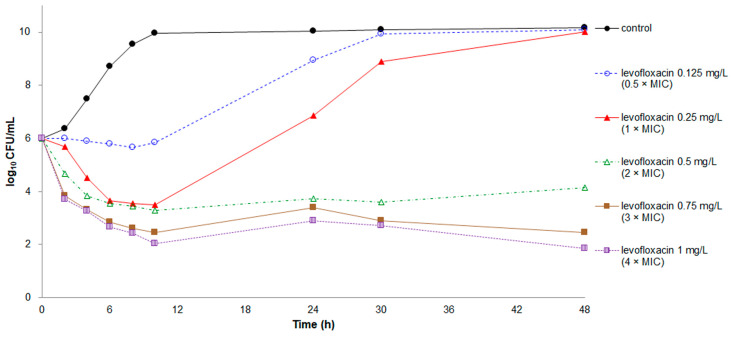
Time-kill assays for levofloxacin against *E. anophelis* strain ED853-49. Data points represent the mean of three experiments. MIC of levofloxacin = 0.25 mg/L.

Rapid killing effects were recorded at all tested concentrations of ciprofloxacin and levofloxacin except for levofloxacin at the concentration of 0.5 × MIC (Figure 3 and Figure 4). Nevertheless, regrowth occurred in *E. anophelis* treated with ciprofloxacin, regardless of the concentration, and levofloxacin at concentrations of 0.5–1 × MIC. Both ciprofloxacin and levofloxacin at concentrations of 3 × and 4 × MIC demonstrated a bactericidal effect, with a CFU number decrease of ≥3 log_10_.

### 3.3. Time-Kill Studies of Dual-Agent Combinations

**Figure 5:**
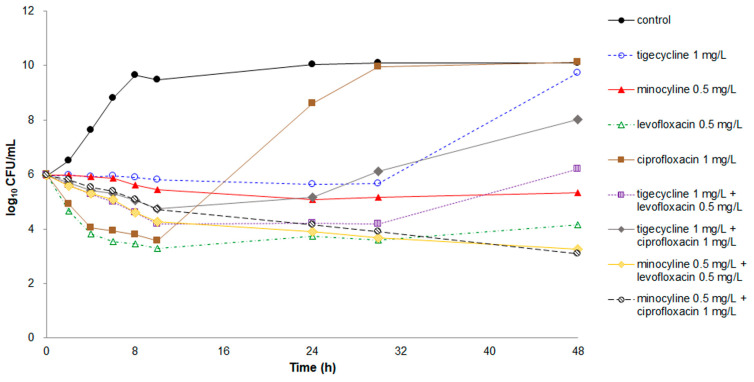
Time-kill assays for single and dual-agent combinations with 2× MIC against *E. anophelis* strain ED853-49. Data points represent the mean of three experiments. Minocycline: MIC, 0.25 mg/L; tigecycline: MIC, 0.5 mg/L; ciprofloxacin: MIC, 0.5 mg/L; and levofloxacin: MIC, 0.25 mg/L.

When minocycline or tigecycline was combined with either ciprofloxacin or levofloxacin (Figure 5), the killing ability of the antibiotics became weaker than that of ciprofloxacin or levofloxacin alone during the first 10 h. However, the reduction in killing activity was <2 log_10_ CFU. Regrowth of bacteria occurred after the 30-h inhibitory period for the tigecycline–ciprofloxacin and tigecycline–levofloxacin combinations. The CFU counts of the tigecycline–levofloxacin combination at 48 h were higher than that of levofloxacin alone.

The minocycline–ciprofloxacin and minocycline–levofloxacin combinations achieved the lowest CFU counts at 48 h. The difference in CFU counts at 48 h for *E. anophelis* inoculated between the minocycline–ciprofloxacin combination and minocycline alone was 2.2 log_10_, meeting the criteria of synergy. Although a desirable synergistic effect was not observed, increased antimicrobial efficacy with a CFU number decrease of 0.9 log_10_ at 48 h was detected in the minocycline–levofloxacin combination compared with levofloxacin alone.

### 3.4. Therapeutic Effects of Antibiotics in the Zebrafish Animal Model

**Figure 6:**
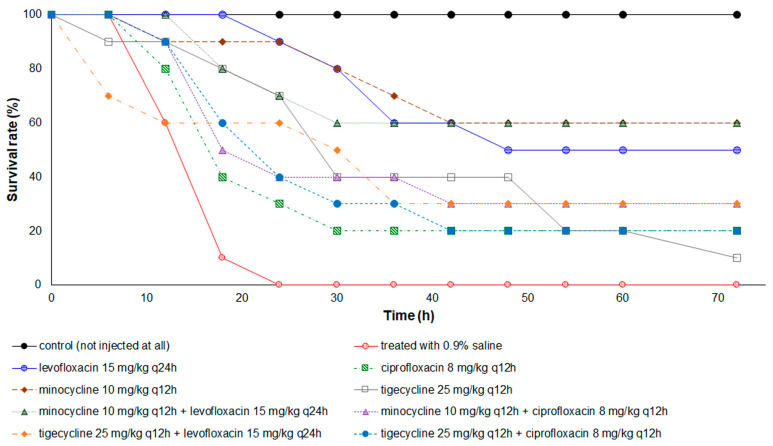
Kaplan–Meier survival curves of the 72-h cumulative survival rate for zebrafish infected with *E. anophelis* strain ED853-49. All zebrafish treated with 0.9% saline died within 24 h. Zebrafish treated with antibiotic(s) exhibited a significantly higher survival rate than those treated with 0.9% saline (*p* < 0.005). The log rank test: Minocycline versus tigecycline, *p* = 0.035; levofloxacin + minocycline versus tigecycline, *p* = 0.049; and others, no significant difference.

With an inoculum of *E. anophelis* via intraperitoneal administration, all zebrafish without antimicrobial treatment died within 24 h (Figure 6). Zebrafish treated with minocycline or minocycline-levofloxacin combination had the highest survival rate (70%) at the end point of 72 h, whereas those treated with tigecycline possessed the lowest survival rate (10%). The median survival time of zebrafish with minocycline or minocycline-levofloxacin combination therapy was >72 h, but for those with ciprofloxacin or minocycline-ciprofloxacin combination treatment, it was only 18 h. The Kaplan–Meier survival analysis revealed that zebrafish treated with all antibiotics, either singly or in combinations, had a significantly higher survival rate than the control group with saline injection. The survival time of the minocycline or minocycline–levofloxacin combination-treated group was significantly longer than that of the tigecycline-treated group (*p* = 0.035 and *p* = 0.049, respectively).

## 4. Discussion

Minocycline, a semi-synthetic tetracycline, is primarily a bacteriostatic antimicrobial agent, and it exerts its antimicrobial activity through the inhibition of protein synthesis [22]. In antimicrobial susceptibility studies using MIC testing [3,4,5,6,7,8], minocycline has been demonstrated as the most active antibiotic against *E. anophelis*. Our study also demonstrated that monotherapy of minocycline exhibited an excellent therapeutic effect in healthy zebrafish infected with *E. anophelis*. However, minocycline expressed a prolonged but slight inhibitory effect rather than a bactericidal effect on *E. anophelis*, even at high concentrations. Because *E. anophelis* usually infects immunocompromised patients [3,4,5,6,7,8], the lack of rapid bacterial killing activity could be a critical problem in the clinical setting.

Tigecycline is an analogue of minocycline and has an extended spectrum to overcome resistance to tetracyclines [23]. In previous antimicrobial susceptibility studies using MIC testing, the susceptibility rate of *E. anophelis* to tigecycline ranged from 5.1% to 26.4% [7]. Although tigecycline is mainly considered to be a bacteriostatic antimicrobial agent [23], this new glycylcycline antibiotic exhibits bactericidal activity against some bacterial species, including *Klebsiella pneumoniae*, *Acinetobacter baumannii*, and *Vibrio vulnificus* [24,25,26]. However, our study revealed that tigecycline possessed only bacteriostatic activity against *E. anophelis* but no bactericidal effect, even at 4 × MIC. In contrast to the bacteriostatic effect of minocycline and tigecycline, both ciprofloxacin and levofloxacin demonstrated rapid bactericidal effects during the first 10 h after initial inoculation. This bactericidal effect is critical since rapid reduction of the bacterial burden in the beginning phase of infections could stabilize the septic condition of patients [27].

Previous studies using time-kill assays have demonstrated in vitro synergistic or antagonistic interactions between tetracycline/glycylcycline and fluoroquinolones against certain microorganisms. For example, the combination of tigecycline and ciprofloxacin was reported to have in vitro synergic effects against *V. vulnificus*, *K. pneumoniae*, and *E. coli* [28,29]. In vitro antagonism occurred in the tigecycline–levofloxacin combination against *Brucella melitensis* [30], but this antagonistic effect was not observed in the treatment of *A. baumannii*, *K. pneumoniae*, or other pathogens [31]. Another study revealed synergistic effects in the tigecycline–levofloxacin combination in the treatment of *A. baumannii* using both chequerboard and time-kill assays [32]. Moreover, the use tigecycline in combination with levofloxacin could produce postantibiotic effects along with enhancement of bactericidal activity and synergistic interaction against *A. baumannii* [33]. Consequently, combination therapy could be a potentially better choice for multidrug-resistant pathogens.

In the present study, the combination of minocycline or tigecycline with ciprofloxacin or levofloxacin markedly reduced the killing effects of ciprofloxacin and levofloxacin on *E. anophelis* in the first 10 h, although this decreased effect did not reach the criteria of antagonism. In addition, minocycline combined with ciprofloxacin or levofloxacin displayed additive bacterial killing activity on *E. anophelis* in the time-kill assays, although only the minocycline–ciprofloxacin combination achieved the criteria for synergy. However, the in vivo animal study revealed that the minocycline–levofloxacin combination exhibited a significantly higher survival rate than the minocycline–ciprofloxacin combination. Our time-kill study demonstrated that rapid regrowth of bacteria occurred after 10 h of killing effects in ciprofloxacin at the concentration of 2 × MIC, but this phenomenon was not observed in levofloxacin at 2 × MIC. This difference could explain why the minocycline–levofloxacin combination demonstrated the best therapeutic effect in zebrafish infected with *E. anophelis*.

Although this study provided valuable information about antimicrobial effects of minocycline, tigecycline, ciprofloxacin, and levofloxacin against *E. anophelis*, it has several limitations. First, only one strain of *E. anophelis* was examined because the time-kill assay is a very labor-intensive and time-consuming task. These results might not represent the antimicrobial effects of these four antibiotics against *E. anophelis*. Second, only four antibiotics were tested in this study. Other potentially effective antibiotics, such as rifampin, trimethoprim/sulfamethoxazole, and vancomycin, were not evaluated. Finally, zebrafish are non-mammal animals. Despite the similar organs and immune system to humans, further experiments using mammal animals might be necessary.

The results of this in vitro and in vivo study suggest that the combination of minocycline and levofloxacin is the most effective therapy for *E. anophelis* infection. Further clinical studies are warranted to delineate the antimicrobial effects of minocycline–levofloxacin combination against this life-threatening infection.
